# TyG Index and Related Indices Predicting Hypertension: Mediation by Neutrophil-to-Lymphocyte Ratio in Multiple Chinese Cohorts

**DOI:** 10.3390/nu17172859

**Published:** 2025-09-03

**Authors:** Mengwen Sun, Yuanyuan Huang, Na Luo, Jinkai Qiu, Yuxuan Lin, Yan Huang, Xiaofeng Zheng, Weihong Qiu, Shanshan Du, Weimin Ye, Heng-Gui Chen

**Affiliations:** 1 Department of Preventive Medicine, The Key Laboratory of Environment and Health, School of Public Health, Fujian Medical University, Fuzhou 350122, China; mengwensun2001@163.com (M.S.); 15306029294@163.com (Y.L.); h_uangyan@126.com (Y.H.); 2 Department of Public Health, Fujian Normal University Hospital, Fujian Normal University, Fuzhou 350007, China; huangyuanyuan@fjnu.edu.cn; 3 Department of Epidemiology and Health Statistics, School of Public Health, Fujian Medical University, Fuzhou 350122, China; ln2248149750@163.com (N.L.); qjk25743@163.com (J.Q.); 13665017197@163.com (X.Z.); whqiu@fjmu.edu.cn (W.Q.); dushanshan1007@163.com (S.D.); 4 Department of Medical Epidemiology and Biostatistics, Karolinska Institute, 171 77 Stockholm, Sweden

**Keywords:** triglyceride-glucose index, hypertension, neutrophil-to-lymphocyte ratio, cohort study

## Abstract

**Background:** Hypertension remains a leading cause of cardiovascular morbidity and mortality globally, and insulin resistance (IR) and systemic inflammation are implicated in the pathogenesis of hypertension. Limited evidence exists on the predictive role of the triglyceride-glucose (TyG) index and its related indices (TyG-WHtR and TyG-WC) for hypertension. This study aimed to investigate these associations across multiple Chinese cohorts. **Methods:** Data from 31,224 participants (Fuqing, CHNS, CHARLS) were analyzed. TyG indices were calculated using fasting triglycerides, glucose, and anthropometrics. Hypertension was defined as SBP/DBP ≥ 140/90 mmHg, or physician diagnosis, or antihypertensive treatment. Logistic/Cox regression models were used to examine associations, adjusting for demographics, lifestyle, and metabolic factors. Mediation analysis quantified the role of neutrophil-to-lymphocyte ratio (NLR) in mediating the TyG–hypertension relationship. **Results:** Elevated TyG index and its obesity-adjusted variants consistently predicted incident hypertension across cohorts (all *p* < 0.001). Each 1-unit TyG increase was associated with 9–36% higher odds of hypertension in Fuqing (OR = 1.09–1.36). NLR mediated 20.4–29.4% of these associations (*p* < 0.001). Subgroup analyses revealed effect modifications by age, sex, and residence. Sensitivity analyses confirmed robustness when redefining hypertension thresholds (ACC/AHA criteria). **Conclusions:** TyG index and its related indices are robust predictors of (new-onset) hypertension, with NLR statistically accounting for approximately 25% of these associations in the mediation model. These findings underscore the interplay between metabolic dysregulation, inflammation, and hypertension and advocate for integrated biomarker strategies in risk stratification and prevention, while external validation in multi-ethnic populations is warranted.

## 1. Introduction

Hypertension, as a dominant contributor to global cardiovascular events and all-cause mortality, poses a substantial public health challenge worldwide [1]. In China, the escalating prevalence of hypertension, affecting an estimated one-third of the adult population (approximately 300 million individuals) during 2014–2015, has been driven by demographic aging and lifestyle transition [2,3]. Consequently, the urgent development of effective primary prevention strategies, combined with timely identification of high-risk populations, is crucial for mitigating the accelerating epidemic of hypertension.

Aberrations in lipoprotein metabolism, particularly elevated plasma triglyceride (TG) levels and impaired fasting blood glucose (FBG) regulation, constitute well-documented cardiovascular risk factors, with pronounced implications for hypertensive populations [4,5]. This phenomenon can be mechanistically attributed to insulin resistance (IR) through three primary pathways: (1) inflammation-mediated endothelial dysfunction [6], (2) ectopic angiotensinogen production [7], and (3) hyperinsulinemia-induced overactivation of the renin-angiotensin-aldosterone system [8]. Recently, the triglyceride-glucose (TyG) index, which is calculated by using TG and FBG [9], has been proposed as a surrogate of IR and been shown to correlate with various indices of IR [10,11]. Nevertheless, existing research has predominantly investigated the association of TyG index with the development of prediabetes [12], diabetes mellitus [13], cardiovascular events [14,15], and all-cause mortality [16,17], while insufficient attention has been devoted to exploring its association with incident hypertension. Only three prospective studies reported a positive association between the TyG index and the incidence of hypertension [18,19,20]. However, these available studies [18,19,20] have been constrained by methodological limitations, including reliance on cross-sectional designs, single-center study population, small sample sizes, and failure to evaluate the TyG index as a continuous variable. Moreover, epidemiological studies have shown that anthropometric measures reflecting central obesity, such as waist-to-height ratio (WHtR) and waist circumference (WC), are more strongly associated with significant cardiometabolic risk than traditional measures reflecting overall obesity with body mass index (BMI) [21,22]. Therefore, combining the TyG index with these metrics may provide additional predictive performance in identifying the risk of (new-onset) hypertension.

The neutrophil-to-lymphocyte ratio (NLR), a hematological index derived from routine complete blood counts, has emerged as a significant biomarker of systemic inflammation and immune regulation [23]. Its diagnostic and prognostic utility extends across diverse clinical conditions, including oncological disorders, inflammatory diseases, metabolic syndrome, psychiatric illnesses, and neurodegenerative conditions [24,25,26,27,28]. A landmark population-based study utilizing data from the 1999–2014 National Health and Nutrition Examination Survey (NHANES), involving 32,454 participants, demonstrated significant associations between NLR elevation and all-cause mortality, particularly mortality from cardiovascular disease, cerebrovascular events, and renal complications [29]. Although substantial evidence has established a consistent positive correlation between elevated NLR levels and both the incidence and prevalence of hypertension [30,31], there is a lack of in-depth and systematic exploration of the mediating role of NLR inflammatory markers in influencing (new-onset) hypertension in association with the TyG index.

In this context, we conducted a study utilizing data from the Fuqing Cohort [32], China Health and Nutrition Survey (CHNS) [33], and China Health and Retirement Longitudinal Study (CHARLS) [34] to examine the association between a series of TyG-related indices and (new-onset) hypertension in the general population. This study has important implications in searching for intervenable biomarkers for stratified prevention and targeted treatment to reduce the risk of hypertension.

## 2. Methods

### 2.1. Study Population

The CHARLS, established in 2011, is a comprehensive biennial survey targeting Chinese residents aged 45 and older. CHARLS collects nationally representative data on various topics, including demographics, family, health status, cognition, healthcare, work, retirement, finances, and housing and serves as a vital resource for understanding the socioeconomic landscape and health dynamics among older adults in China [34]. Our study included participants in 2011 as the baseline, and follow-up surveys were repeated in 2018.

**Figure 1:**
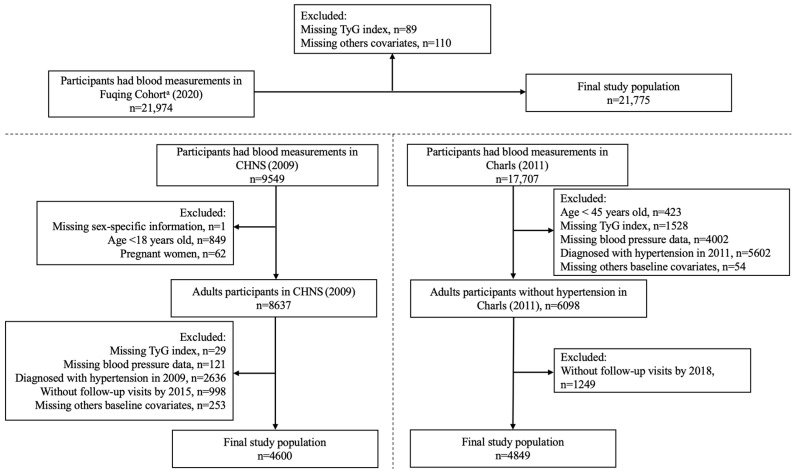
Flowchart of participant selection. ^a^ The Fuqing Cohort survey ended 16 December 2022; TyG, triglyceride-glucose.

As shown in Figure 1, this study included 21,974 participants from the Fuqing Cohort, 9549 participants from CHNS, and 17,707 participants from CHARLS. Individuals were excluded due to missing data on (new-onset) hypertension, TyG index combinations with obesity indicators (including TyG-WC and TyG-WHtR), and demographic data (including age, sex, education level, drinking, smoking, hypertension, total cholesterol, glycosylated hemoglobin A1c (HbA1c), and high-density lipoprotein (HDL)). Finally, our analysis datasets encompassed 21,775 participants, 4600 participants, and 4849 participants from the Fuqing Cohort, CHNS, and CHARLS, respectively.

The Fuqing cohort was the only cohort with data on differential white blood cell counts, allowing for the calculation of the Neutrophil-to-Lymphocyte Ratio (NLR). This is a common occurrence when harmonizing data from independent cohorts designed with different primary objectives and biomarker panels. The CHNS and CHARLS cohorts did not have this data available for the waves included in our analysis.

### 2.2. Assessment of TyG, TyG–WHtR, and TyG–WC

TyG index and its related indices were calculated as follows: TyG index = ln [TG(mg/dL) × FBG(mg/dL)/2], TyG-WHtR index = ln [TG(mg/dL) × FBG(mg/dL)/2] × WC/height, and TyG-WC index = ln [TG(mg/dL) × FBG(mg/dL)/2] × WC [35]. Participants were divided into four groups according to the quartiles of the TyG index and its related indices, with the group in the first quartile serving as the reference.

### 2.3. Outcome

The study outcome was (new-onset) hypertension, defined as mean SBP ≥ 140 mmHg and/or mean DBP ≥ 90 mmHg, or a physician diagnosis of hypertension, or treatment for hypertension according to WHO criteria, whichever came first.

### 2.4. Covariates

Informed by published research and clinical judgment, several variables were identified as potential confounders, including age, sex, residence, education level, smoking and drinking status, BMI, baseline SBP and DBP (except for Fuqing cohort), uric acid, HDL-C, TC, and HbA1c [35,36]. For consistency between the Fuqing Cohort, CHNS, and CHARLS, age was analyzed as a continuous variable, while age was categorized as <65 and ≥65 years in subgroup analyses. Sex was categorized as female and male. Education level was divided into three categories: elementary school, middle school, and high school or above. Self-reported smoking and drinking status were categorized as never and ever (including now and former).

### 2.5. Statistical Analyses

Continuous variables were reported as medians and interquartile ranges (IQR) for non-normal distributions and as means ± standard deviation (SD) for normal distributions, respectively. Categorical variables were presented as frequencies (percentage, %). TyG index and its related indices’ levels were categorized into quartiles for group comparisons. Categorical group differences were assessed using the Pearson Chi-squared test, while continuous variables were compared using one-way ANOVA for normally distributed data and the Kruskal–Wallis test for non-normally distributed data, where appropriate. The logistic regression analysis was conducted to evaluate the association of TyG index and its related indices with hypertension in Fuqing Cohort. The new-onset hypertension incidence rate was expressed as per 1000 person-years. Univariable and multivariable Cox proportional hazards regression models were constructed to assess the association of TyG index and its related indices with new-onset hypertension in CHNS and CHARLS, with results presented as hazard ratios (HRs) and 95% confidence intervals (95% CI). We also performed restricted cubic spline (RCS) Cox regression with 4 knots (5th, 35th, 65th, and 95th percentiles of TyG index and its related indices) to test for nonlinear association and characterize dose–response relationships between TyG index and its related indices and (new-onset) hypertension. Model 1 adjusted for sex, age, education level, residence, BMI, baseline SBP and DBP (except for Fuqing cohort), smoking, and drinking. Model 2 was further adjusted for biochemical variables (including uric acid, HDL-C, TC, and HbA1c). A mediation analysis was carried out to assess the indirect impact of TyG index on hypertension mediated through NLR. The mediated proportion was calculated as the log hazard ratio of the natural indirect effect divided by the log hazard ratio of the total effect [37].

To evaluate potential effect modification by different variables for the association between TyG index and (new-onset) hypertension, participants were stratified by age (<65 vs. ≥65 years), sex, residence (urban vs. rural), education level (elementary school vs. middle school vs. high school or above), FBG (<100 vs. ≥100 mg/dL), and TG (<200 vs. ≥200 mg/dL). We included an interaction term in the model for each analysis to assess effect measure modification. All analyses were performed using Stata 18 and R statistical software (R project, version 4.3.2).

### 2.6. Sensitivity Analysis

The robustness of the results was further verified by various sensitivity analyses. First, we excluded self-reported hypertension (including diagnosis status and anti-hypertensive medication use) from questionnaire-based assessments to address potential recall bias. Second, we reclassified hypertension according to the 2017 American College of Cardiology (ACC)/American Heart Association (AHA) guidelines [38], defining cases as average SBP ≥ 130 mmHg and/or average DBP ≥ 80 mmHg, or a physician hypertension diagnosis, or taking anti-hypertensive medication.

## 3. Results

### 3.1. Baseline Characteristics

The Fuqing Cohort encompasses 21,775 participants, presenting a mean TyG index of 8.4 (SD = 0.7), while the CHNS and CHARLS cohorts consist of 4600 and 4849 participants, with mean TyG indices of 8.6 (SD = 0.7) and 8.5 (SD = 0.7), respectively. The sex distribution exhibited differences across cohorts, ranging from 35.6% male in the Fuqing Cohort to 44.7% male in both the CHNS and CHARLS cohorts. Average blood pressure was highest in the Fuqing Cohort (131.2/83.1 mmHg) and lowest in the CHARLS cohort (119.8/75.1 mmHg). Average BMI levels exhibited variation across cohorts, ranging from 23.3 kg/m^2^ in the CHARLS cohort to 24.1 kg/m^2^ in the Fuqing Cohort. The prevalence of smoking, alcohol drinking, urban residence, and educational attainment further highlighted cohort-specific differences, paralleled by distinct patterns in lipid profiles and uric acid levels (Table 1).

### 3.2. Association Between TyG Index and Its Related Indices and Hypertension

Table 2 presents the odds ratio (OR) of the TyG index and its related indices with hypertension in the Fuqing Cohort and the HRs of the TyG index and its related indices with new-onset hypertension in the CHNS and CHARLS cohorts. For the TyG index, the OR for hypertension and the HRs for new-onset hypertension showed significant graded increases with ascending quartiles across all three cohorts (all *p* for trend < 0.001). Taking the Fuqing Cohort as an example, the crude ORs of the second quartile (Q2), Q3, and Q4 were 1.41, 1.75, and 1.94 when compared with the lowest quartile of the TyG index, respectively. This upward trend persisted after adjusting for multiple factors in Model 1 and Model 2. For each one-unit increase in the TyG index, the OR and HRs in the three cohorts also demonstrated different degrees of increase, indicating that a higher TyG index was associated with an increased risk of (new-onset) hypertension.

Similar trends were observed for the TyG-WHtR and TyG-WC index. In the Fuqing Cohort, the OR for hypertension rose significantly as the quartiles of TyG-WHtR and TyG-WC increased (all *p* for trend < 0.001). For example, for TyG-WHtR in the Fuqing Cohort, the ORs for Q2, Q3, and Q4 compared with Q1 in the crude model were 1.42, 1.83, and 2.34, respectively. For each one-unit increase in TyG-WHtR and each 100.0 increase in TyG-WC, the ORs also increased, suggesting that these two indices were also closely related to the risk of hypertension.

**Figure 2:**
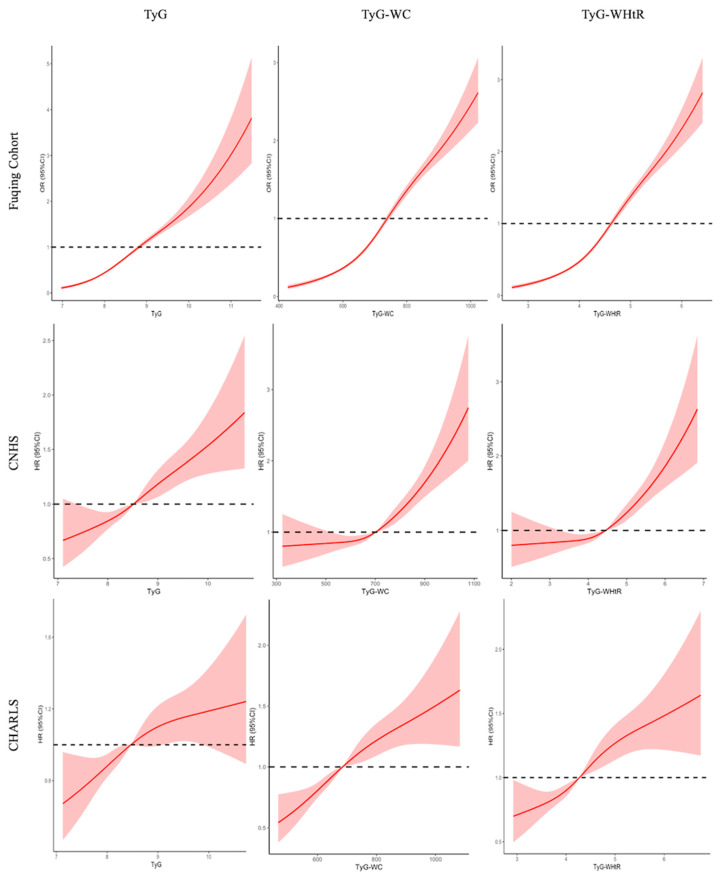
Restricted cubic spline curve for the association of TyG and its related indices with hypertension. Solid lines represent hazard ratios, and dashed lines represent 95% confidence intervals; Adjusted for sex, age, education, urban, baseline SBP and DBP (except for Fuqing cohort), smoking, drinking, uric acid, HDL-C, TC, and HbA1c; The red solid lines represent the effect estimates, and the shadowed parts are 95% confidence intervals; two-piece Cox proportional hazards models were used to estimate the risk inflection point; TyG, triglyceride-glucose; WHtR, waist to height ratio; WC, waist circumference.

Figure 2 illustrates the dose–response associations of TyG index and its related indices with the risk of (new-onset) hypertension using restricted cubic spline curves. The logistic regression model and the Cox proportional hazards models identified inflection points in the risk curves, suggesting nonlinear relationships. Specifically, the risk of hypertension increased progressively with elevated TyG index and its related indices, with steeper gradients observed beyond certain thresholds in Fuqing cohort (TyG: 8.5; TyG-WC: 700; TyG-WHtR: 4.5).

### 3.3. Mediation Analysis of NLR on the TyG–Hypertension Association

Table 3 presents the mediating role of NLR on the association of TyG index and its related indices with hypertension in the Fuqing Cohort. The total effect of TyG on hypertension was 1.67 (95% CI: 1.38–1.98), and NLR mediated 29.4% of this effect (mediated effect: 0.86, 95% CI: 0.80–0.92; *p* < 0.001). Similarly, TyG-WHtR showed a total effect of 1.87 (95% CI: 1.66–2.09), and NLR mediated 20.4% of the association (mediated effect: 0.88, 95% CI: 0.83–0.94; *p* < 0.001). For TyG-WC, the total effect was 1.74 (95% CI: 1.41–2.08), and NLR mediated 29.3% of the association (mediated effect: 0.85, 95% CI: 0.81–0.91; *p* < 0.001).

### 3.4. Subgroups Analysis of the Association Between TyG-Related Indices and Hypertension

**Figure 3:**
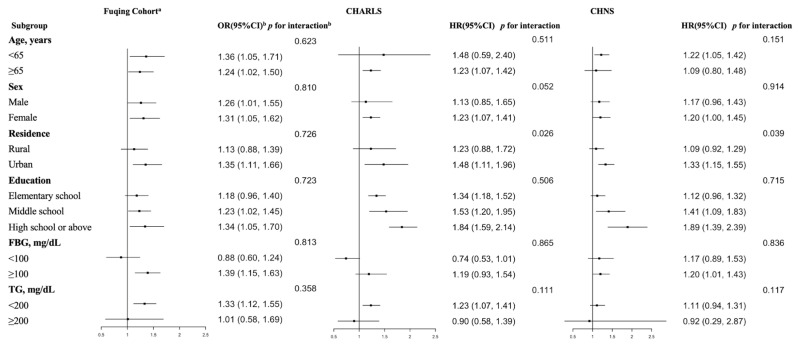
Subgroup analyses of the relationship between TyG index and its related indices and hypertension. ^a^ The Fuqing Cohort survey ends 16 December 2022; ^b^ The odds/hazard ratios were adjusted for sex, age, education, urban, BMI, baseline SBP and DBP (except for Fuqing cohort), smoking, drinking, uric acid, HDL-C, TC, and HbA1c; In this study, wave 8 (in 2009) of CHNS and wave 1 (in 2011) of CHARLS were regarded as the baseline, respectively. Subsequent follow-up surveys were used to follow the outcome until the final follow-up survey, which was wave 10 of CHNS (in 2015) and wave 4 (in 2018) of CHARLS; FBG, fasting blood glucose; TG, triglyceride.

Figure 3 presents the results of subgroup analyses examining the relationship between the TyG index and hypertension across three distinct cohorts. While suggestive trends or marginal differences were observed in several subgroups, most of the interaction effects did not achieve statistical significance. Notably, significant interaction was identified only for the subgroup of place of residence in the CHNS and CHARLS (*p* = 0.039 and 0.026, respectively).

### 3.5. Sensitivity Analyses

The results remained consistent when several methods were utilized to verify the robustness of the relationship between TyG-related indices and hypertension. After excluding the self-reported hypertension, the results were not substantially changed (CHNS: Q2-Q4 vs. Q1, HR, 1.24; 95% CI 1.06–1.45) (Supplement: Tables S6 and S7). Although there was no significant statistical difference, similar trends were also observed between TyG-related indices and hypertension after redefining hypertension by the new ACC and AHA guidelines with a blood pressure threshold of 130/80 (Supplement: Tables S8–S10).

## 4. Discussion

The present study, leveraging data from three large-scale Chinese cohorts, provides robust evidence that the TyG index and its related indices were significant predictors of (new-onset) hypertension. Furthermore, our mediation analysis revealed that systemic inflammation, as quantified by the NLR, mediated approximately 20.4–29.4% of the TyG–hypertension association. These findings advanced our understanding of the interplay between metabolic dysregulation, chronic inflammation, and hypertension pathogenesis, offering novel insights into risk stratification and preventive strategies. Below, we contextualize these results within the existing literature, explore their mechanistic implications, and discuss their clinical and public health relevance.

### 4.1. Key Findings in Context of Existing Evidence

Our results align with prior studies highlighting the TyG index as a surrogate marker of insulin resistance (IR) and its association with cardiometabolic diseases. For instance, Sanchez-Inigo et al. [18] reported a positive correlation between TyG index and hypertension risk in a Spanish cohort, and Zheng and Mao [19] also demonstrated similar trends in a Chinese population. However, our study extends these findings by incorporating obesity-adjusted indices and revealing their superior predictive performance compared to the TyG index alone. Our study resonates with recent work by Dang et al. [35], who emphasized the enhanced discriminative power of combining TyG with central obesity measures for cardiovascular risk assessment.

The mediating role of NLR in the TyG–hypertension relationship represents a novel contribution to the field. While previous studies have independently linked elevated NLR to hypertension [30,31] and IR [27], recent evidence further establishes NLR as a key biomarker of endothelial dysfunction and vascular pathology across diverse clinical contexts [39]. Our mediation models quantitatively indicate that NLR could represent a biologically plausible pathway linking TyG indices to hypertension, though causality requires experimental validation. This finding aligns with emerging paradigms of immunometabolic crosstalk in hypertension pathogenesis, reinforced by NLR’s robust translational value in vascular diseases ranging from carotid stenosis to peripheral arterial pathology [40]. Specifically, IR-driven metabolic dysregulation is increasingly understood to propagate through inflammatory pathways. Hyperinsulinemia and dyslipidemia can activate innate immune cells, leading to the release of pro-inflammatory cytokines such as IL-1β, IL-6, and TNF-α, which in turn promote endothelial dysfunction, vascular stiffness, and sodium retention—key hallmarks of hypertension [41,42]. Furthermore, dietary factors, particularly high intake of sodium, sugar-sweetened beverages, and ultra-processed foods, have been recently shown to exacerbate both IR (elevating TyG) and systemic inflammation (elevating NLR), creating a synergistic pathway towards hypertension [43,44]. This suggests that the TyG–NLR–hypertension axis we observed may be potentiated by modern dietary patterns. Thus, while our mediation analysis indicates that NLR represents a biologically plausible pathway linking TyG indices to hypertension, this pathway is likely embedded within a broader network of dietary and immunometabolic interactions, and causality requires further experimental validation.

Moreover, the interpretation of NLR as a mediator warrants caution. While our statistical model suggests a significant mediating effect, it is plausible that NLR is not itself a direct causal agent but rather a surrogate marker for other unmeasured pathophysiological processes linked to both insulin resistance and hypertension. For instance, renal dysfunction, which is closely associated with both IR and systemic inflammation, can elevate NLR and contribute to hypertension through fluid retention and RAAS (Renin-Angiotensin-Aldosterone System) activation. Similarly, expanded and dysfunctional adipose tissue, particularly visceral fat, is a potent source of pro-inflammatory cytokines (e.g., IL-6, TNF-α) and adipokines (e.g., leptin) that can drive both neutrophilia and lymphopenia, thus elevating NLR, while also promoting endothelial dysfunction and vascular remodeling through direct mechanisms [45,46]. Furthermore, NLR may reflect low-grade inflammation originating from other sources, such as the gut microbiota or subclinical infections, which have been increasingly implicated in metabolic and cardiovascular diseases [47]. Therefore, although NLR operationalizes a part of the inflammatory pathway in our analysis, it likely serves as an integrative, albeit non-specific, biomarker of a complex network of immunometabolic disturbances. Future research employing more specific inflammatory panels (e.g., IL-1β, IL-6, TNF-α) and incorporating measures of renal function and body composition will be crucial to disentangle the precise biological pathways that NLR represents in this context.

### 4.2. Clinical and Public Health Implications

Our findings have immediate implications for risk stratification and primary prevention. First, the TyG index and its related indices are simple, cost-effective biomarkers that can be easily calculated using routine clinical measurements (e.g., triglycerides, glucose, and WC). Recent studies have begun to integrate these indices into validated risk prediction models for cardiovascular diseases, showing incremental value beyond traditional risk factors [48]. Integrating these indices into hypertension screening protocols could enhance the early identification of high-risk individuals, particularly in resource-limited settings where advanced diagnostic tools are unavailable. The NLR, as a ubiquitous hematological parameter, further adds a layer of inflammatory risk assessment that is readily available at no additional cost, strengthening the case for its use in composite risk scores.

Second, the mediating role of NLR suggests that anti-inflammatory therapies could complement existing antihypertensive strategies. While lifestyle changes (e.g., Mediterranean diet, and weight loss) reduce both IR and inflammation [49], there is growing interest in the anti-inflammatory effects of specific pharmacological agents. Sodium-glucose cotransporter 2 (SGLT2) inhibitors have been shown to attenuate NLR and reduce IL-6 levels, partly independent of their glycemic effects [50,51]. Similarly, interleukin-1β (IL-1β) antagonists have shown promise in improving vascular function and reducing cardiovascular events in patients with heightened inflammation [52,53]. Our data suggest that patients with elevated TyG indices might be particularly good candidates for such inflammation-targeting therapies, a hypothesis worthy of future investigation in randomized trials.

At the public health level, our results advocate for policies addressing the dual epidemics of metabolic syndrome and chronic inflammation. Our findings, combined with recent literature [43,44], underscore the importance of dietary quality beyond just caloric intake. For instance, policies aimed at reducing population-level consumption of ultra-processed foods and sugar-sweetened beverages (major sources of refined sugars and unhealthy fats) and promoting potassium-rich foods to counterbalance sodium intake could simultaneously mitigate IR and systemic inflammation. Additionally, promoting urban green spaces to encourage physical activity remains a crucial strategy to disrupt the IR–inflammation–hypertension axis at the community level.

### 4.3. Strengths and Limitations

Our study utilized three large-scale prospective Chinese cohorts with 31,224 participants to establish a robust multi-cohort framework, enhancing generalizability and replicability. By innovatively integrating the TyG index and its related indices, it enables comprehensive assessments of systemic metabolic dysregulation and visceral adiposity, offering a multidimensional approach for cardiometabolic risk stratification. Mechanistically, our study identified the mediating role of inflammation (quantified by NLR) in the TyG–hypertension pathway for the first time, elucidating the interplay between metabolic dysfunction and chronic inflammation. Finally, the identification of modifiable biomarkers (TyG index and NLR) and mechanistic insights provides actionable targets for early hypertension risk prediction and intervention strategies, aligning with global priorities to mitigate cardiovascular disease burden.

However, limitations must be acknowledged. First, selection bias may be present. Participants with complete data included in the final analysis may systematically differ from those excluded due to missing values, potentially limiting the representativeness of our sample and the internal validity of our findings. Second, potential measurement biases should be considered. Although we used a combination of measured blood pressure and self-report to define hypertension, self-reported physician diagnoses and medication use are subject to recall bias. Furthermore, key biomarkers, including NLR, were assessed at a single time point, which may not reflect long-term exposure levels and precludes the analysis of temporal changes. The lack of repeated measures also means we could not account for regression dilution bias, potentially attenuating the estimated effect sizes toward the null. Third, the observational design precludes definitive causal conclusions. Although we used prospective data and performed sensitivity analyses to mitigate reverse causation, the possibility of unmeasured confounding remains. The mediation analysis, while suggestive, highlights statistical pathways that require validation through experimental studies to establish causality. Fourth, although mediation analysis suggests that NLR may partially explain the association between TyG indices and hypertension, the observational design and potential unmeasured confounders—particularly dietary factors such as sodium intake—preclude definitive causal conclusions. Sodium intake is a well-established and strong determinant of hypertension, and its omission is a central limitation given China’s regional heterogeneity in salt consumption and the interrelationships among diet, metabolic markers, and inflammation. Despite extensive adjustment for known demographic, lifestyle, and metabolic covariates, residual confounding cannot be fully ruled out. Therefore, our findings primarily highlight statistical associations and generate hypotheses for mechanistic research, while we recommend cautious interpretation of the results and encourage future studies to incorporate detailed dietary assessments to better clarify these relationships. Finally, our study population was restricted to Chinese adults, limiting direct extrapolation to other ethnicities. Genetic, lifestyle, and environmental factors vary substantially across populations and may modify the TyG–hypertension relationship. Future studies in multi-ethnic cohorts are essential to validate our findings and assess ethnic-specific risk stratification strategies.

## 5. Conclusions

In conclusion, our multi-cohort study establishes the TyG index and its related indices as robust predictors of hypertension, with systemic inflammation partially mediating these relationships. These findings underscore the importance of addressing both metabolic and inflammatory pathways in hypertension prevention. Future studies must validate these associations across diverse ethnic groups to translate biomarker-driven strategies globally.

## Figures and Tables

**Table 1:** Baseline characteristics of eligible participants in three cohorts.

Variables	Fuqing Cohort	CHNS	CHARLS
*n*	21,775	4600	4849
TyG index	8.4 (0.7)	8.6 (0.7)	8.5 (0.7)
TyG-WHtR	4.4 (3.8, 5.0)	4.5 (4.0, 5.0)	4.5 (4.0, 5.0)
TyG-WC	724.8 (648.4, 814.9)	747.1 (671.6, 830.2)	735.3 (660.5, 818.2)
Age, years (mean ± SD)	56.7 (10.0)	48.1 (13.6)	58.8 (9.1)
Male (%)	7747 (35.6)	2058 (44.7)	2167 (44.7)
SBP, mmHg	131.2 (20.0)	116.4 (11.2)	119.8 (11.2)
DBP, mmHg	83.1 (11.1)	76.0 (7.5)	75.1 (7.4)
BMI, kg/m^2^	24.1 (3.3)	22.9 (3.2)	23.3 (1.7)
Smoking, *n* (%)	5917 (27.2)	1286 (28.0)	1462 (30.2)
Alcohol drinking, *n* (%)	1973 (9.1)	1480 (32.2)	1589 (32.8)
Urban residence, *n* (%)	4242(19.5)	1335 (29.0)	4038 (83.3)
Education, *n* (%)			
Elementary school	14,847 (68.2)	1864 (40.5)	2977 (61.4)
Middle school	4926 (22.6)	2169 (47.2)	1189 (24.5)
High school or above	2002 (9.2)	567 (12.3)	683 (14.1)
TG, mmol/L	1.4 (0.8, 1.8)	1.2 (0.8, 1.8)	1.2 (0.8, 1.7)
FBG, mmol/L	5.4 (1.6)	5.3 (1.3)	5.7 (1.2)
TC, mmol/L	5.7 (5.0, 6.4)	4.7 (4.1, 5.4)	4.2 (3.5, 5.1)
HDL-C, mmol/L	1.6 (1.3, 1.8)	1.4 (1.2, 1.6)	1.3 (1.0, 1.6)
Uric acid, μmol/L	352.6 (304.2, 413.5)	284.0 (231.0, 346.3)	297.0 (245.3, 359.6)
HbA1c, %	5.4 (0.9)	5.5 (0.8)	5.5 (0.8)
NLR	1.7 (0.8)	-	-

Abbreviation: TyG, triglyceride-glucose; BMI, body mass index; WC, waist circumference; WHtR, waist to height ratio; SBP, systolic blood pressure; DBP, diastolic blood pressure; TG, triglyceride; FBG, fasting blood glucose; TC, total cholesterol; HDL-C, high density lipoprotein cholesterol; HbA1c, glycosylated hemoglobin A1c; NLR, neutrophil to lymphocyte ratio.

**Table 2:** The odds/hazard ratios of TyG index and its related indices with hypertension.

Variables	Fuqing Cohort, OR (95% CI)			CHNS, HR (95% CI)			CHARLS, HR (95% CI)		
	Crude	Model 1	Model 2	Crude	Model 1	Model 2	Crude	Model 1	Model 2
	Model			Model			Model		
**Quartiles of TyG index**									
Q1	Ref.	Ref.	Ref.	Ref.	Ref.	Ref.	Ref.	Ref.	Ref.
Q2	1.41	1.20	1.25	1.45	1.27	1.29	1.59	1.35	1.29
	(1.17–1.69)	(1.02–1.53)	(1.04–1.50)	(1.22–1.74)	(1.06–1.52)	(1.07–1.55)	(1.35–1.87)	(1.13–1.61)	(1.07–1.55)
Q3	1.75	1.41	1.42	1.62	1.23	1.24	1.73	1.62	1.54
	(1.55–1.98)	(1.25–1.68)	(1.21–1.65)	(1.37–1.93)	(1.03–1.47)	(1.03–1.49)	(1.50–2.01)	(1.36–1.92)	(1.33–1.79)
Q4	1.94	1.55	1.57	2.21	1.47	1.5	2.87	2.96	2.5
	(1.40–2.67)	(1.28–2.03)	(1.13–2.03)	(1.86–2.62)	(1.23–1.76)	(1.22–1.84)	(2.46–3.35)	(2.45–3.57)	(2.22–2.84)
*p* for trend	<0.001	<0.001	<0.001	<0.001	<0.001	<0.001	<0.001	<0.001	<0.001
Per 1.0 increase	1.36	1.14	1.09	1.39	1.14	1.17	1.06	1.07	1.07
	(1.09–1.70)	(1.05–1.29)	(1.03–1.27)	(1.29–1.50)	(1.05–1.24)	(1.04–1.31)	(1.05–1.07)	(1.06–1.08)	(1.06–1.08)
**Quartiles of TyG-WHtR index**									
Q1	Ref.	Ref.	Ref.	Ref.	Ref.	Ref.	Ref.	Ref.	Ref.
Q2	1.42	1.16	1.19	1.16	1.16	1.13	1.81	1.46	1.43
	(1.26–1.61)	(0.98–1.39)	(1.02–1.44)	(0.93–1.46)	(0.92–1.46)	(0.90–1.42)	(1.54–2.13)	(1.22–1.75)	(1.20–1.72)
Q3	1.83	1.60	1.65	1.93	1.93	1.82	2.31	1.83	1.82
	(1.57–2.12)	(1.38–1.80)	(1.44–1.87)	(1.57–2.37)	(1.57–2.37)	(1.48–2.24)	(1.98–2.69)	(1.51–2.22)	(1.47–2.21)
Q4	2.34	2.01	2.10	2.31	2.33	2.18	3.33	2.78	2.78
	(2.09–2.63)	(1.73–2.40)	(1.82–2.41)	(1.89–2.82)	(1.91–2.85)	(1.78–2.66)	(2.83–3.92)	(2.26–3.42)	(2.28–3.45)
*p* for trend	<0.001	<0.001	<0.001	<0.001	<0.001	<0.001	<0.001	<0.001	<0.001
Per 1.0 increase	1.74	1.22	1.22	1.74	1.75	1.69	1.28	1.27	1.26
	(1.34–2.27)	(1.14–1.34)	(1.05–1.50)	(1.37–2.20)	(1.38–2.21)	(1.33–2.15)	(1.24–1.31)	(1.22–1.32)	(1.21–1.31)
**Quartiles of TyG-WC index**									
Q1	Ref.	Ref.	Ref.	Ref.	Ref.	Ref.	Ref.	Ref.	Ref.
Q2	1.5	1.23	1.21	1.26	1.26	1.21	1.74	1.45	1.42
	(1.25–1.81)	(1.05–1.51)	(1.05–1.39)	(0.97–1.64)	(0.96–1.63)	(0.93–1.58)	(1.46–2.08)	(1.17–1.80)	(1.13–1.78)
Q3	1.88	1.55	1.53	1.69	1.7	1.6	2.3	1.97	1.89
	(1.49–2.38)	(1.21–1.95)	(1.20–1.87)	(1.32–2.17)	(1.33–2.18)	(1.25–2.05)	(1.91–2.77)	(1.55–2.52)	(1.45–2.38)
Q4	2.39	2.00	1.96	2	2.04	1.89	2.95	2.76	2.69
	(1.95–3.05)	(1.62–2.52)	(1.54–2.48)	(1.62–2.62)	(1.60–2.60)	(1.48–2.41)	(2.42–3.60)	(2.13–3.57)	(2.08–3.51)
*p* for trend	<0.001	<0.001	<0.001	<0.001	<0.001	<0.001	<0.001	<0.001	<0.001
Per 100.0 increase	1.62	1.19	1.18	1.52	1.55	1.48	1.23	1.24	1.24
	(1.31–2.00)	(1.05–1.33)	(1.03–1.32)	(1.32–1.75)	(1.34–1.78)	(1.28–1.71)	(1.18–1.27)	(1.19–1.30)	(1.13–1.37)

Model 1: adjusted for sex, age, education, urban, BMI, baseline SBP and DBP (except for Fuqing cohort), smoking, and drinking; Model 2 (Full model): Model 1 and further adjusted for uric acid, HDL-C, TC, and HbA1c; Abbreviation: TyG, triglyceride-glucose; WHtR, waist to height ratio; WC, waist circumference; Q, quartile; HR, hazard ratio; CI, confidence interval; Ref., Reference.

**Table 3:** The mediating role of NLR on the association between TyG index and its related indices and hypertension in Fuqing Cohort.

Outcome	Independent Variable	Total Effect (95% CI)	Mediated Effect of NLR (95% CI)	*p* Value	Estimated Proportion Mediated
Hypertension	TyG	1.67 (1.38–1.98)	0.86 (0.80–0.92)	0.001	29.4%
	TyG-WHtR	1.87 (1.66–2.09)	0.88 (0.83–0.94)	0.001	20.4%
	TyG-WC	1.74 (1.41–2.08)	0.85 (0.81–0.91)	0.001	29.3%

Models were adjusted for sex, age, education, urban, BMI, smoking, drinking, uric acid, HDL-C, TC and HbA1c; Abbreviation: TyG, triglyceride-glucose; WHtR, waist to height ratio; WC, waist circumference; CI, confidence interval; NLR, neutrophil to lymphocyte ratio.

## Data Availability

Data will be made available upon reasonable request to the corresponding authors.

## Supplementary Materials

The following supporting information can be downloaded at: https://www.mdpi.com/article/10.3390/nu17172859/s1, Table S1: Distribution of missing variables. Table S2: Baseline characteristics of excluded and included participants in CHNS. Table S3: Baseline characteristics of excluded and included participants in CHARLS. Table S4: Baseline characteristics of participants stratified by outcome in CHNS. Table S5: Baseline characteristics of participants stratified by outcome in CHARLS. Table S6: The association of TyG index and its related indices with hypertension after excluding the questionnaire data defining hypertension in Fuqing Cohort. Table S7: The association of TyG index and its related indices with new-onset hypertension after excluding the questionnaire data defining hypertension in CHNS. Table S8: The association of TyG index and its related indices with hypertension defined by the novel diagnostic criteria (SBP/DBP: 130/80) in Fuqing Cohort. Table S9: The association of TyG index and its related indices with new-onset hypertension defined by the novel diagnostic criteria (SBP/DBP: 130/80) in CNHS. Table S10: The association of TyG index and its related indices with new-onset hypertension defined by the novel diagnostic criteria (SBP/DBP: 130/80) in CHARLS.
