# Supplementary material for: TyG Index and Related Indices Predicting Hypertension: Mediation by Neutrophil-to-Lymphocyte Ratio in Multiple Chinese Cohorts
Source: Nutrients. 2025 Sep 3;17(17):2859. doi: 10.3390/nu17172859 (PMC12430566; doi:10.3390/nu17172859)
Supplement: Supplementary file 1 [file nutrients-17-02859-s001.zip › nutrients-3839557-supplementary.pdf]

Supplemental data

Supplemental Table S1 Distribution of missing variables.

Characteristic	Number of participants in Fuqing Cohort (n=199)	Number of participants in CHNS (n=176)	Number of participants in CHARLS (n=54)
BMI	143	31	15
WHtR	25	86	27
HbA1c	0	26	4
Hemoglobin	0	12	0
LDL-C	18	6	0
Smoking status	5	2	1
Drinking status	4	1	0
Education	4	12	7

BMI, body mass index; WHtR, waist to height ratio; HbA1c, hemoglobin A1c; LDL-C, low density lipoprotein cholesterol.

Supplemental Table S2 Baseline characteristics of excluded and included participants in CHNS.

Variables	Overall	Excluded	Included	*P* value
n	9549	4949	4600	
TyG index	8.6 (0.7)	8.6 (0.8)	8.6 (0.7)	<0.001
TyG-WHtR	4.5 (3.9, 5.0)	4.4 (3.8, 5.1)	4.5 (4.0, 5.0)	<0.001
TyG-WC	750.8 (670.6, 834.1)	755.5 (669.4, 842.0)	747.1 (671.6, 830.2)	<0.001
Age, years old	46.8 (18.2)	45.6 (21.6)	48.1 (13.6)	<0.001
Male (%)	4539 (47.5)	2481 (50.1)	2058 (44.7)	<0.001
SBP, mmHg	122.7 (19.9)	128.9 (24.1)	116.4 (11.2)	<0.001
DBP, mmHg	79.4 (11.9)	82.7 (14.1)	76.0 (7.5)	<0.001
BMI, kg/m²	22.9 (3.8)	22.9 (4.4)	22.9 (3.2)	0.581
Smoking, n (%)	2395 (26.4)	1109 (24.7)	1286 (28.0)	0.001

Drinking, n (%)	2858 (31.5)	1378 (30.7)	1480 (32.2)	0.147
**Education, n (%)**				<0.001
Illiteracy	2320 (24.5)	1383 (28.4)	937 (20.4)	
Primary school	1962 (20.7)	1035 (21.2)	927 (20.2)	
Middle school	4142 (43.7)	1973 (40.5)	2169 (47.2)	
High school or above	1053 (11.1)	486 (10.0)	567 (12.3)	
Triglycerides, mmol/L	1.2 (0.8, 1.9)	1.3 (0.8, 2.0)	1.2 (0.8, 1.8)	0.001
TC, mmol/L	4.7 (4.1, 5.4)	4.7 (4.0, 5.4)	4.7 (4.1, 5.4)	0.042
LDL-C, mmol/L	2.8 (2.3, 3.5)	2.8 (2.2, 3.5)	2.9 (2.3, 3.5)	0.003
HbA1c, %	5.6 (0.9)	5.7 (1.0)	5.5 (0.8)	<0.001
FBG, mmol/L	5.4 (1.4)	5.4 (1.5)	5.3 (1.3)	<0.001
Uric acid, μmol/L	296.0 (238.0, 362.0)	306.0 (246.0, 373.0)	284.0 (231.0, 346.3)	<0.001

Q, quartile; TyG, triglyceride-glucose; WHtR, waist to height ratio; WC, waist circumference; SBP, systolic blood pressure; DBP, diastolic blood pressure; BMI, body mass index; TC, total cholesterol; LDL-C, low density lipoprotein cholesterol; HbA1c, glycosylated hemoglobin A1c; FBG, fasting blood glucose.

Supplemental Table S3 Baseline characteristics of excluded and included participants in CHARLS.

Variables	Overall	Excluded	Included	*P* value
n	17707	12858	4849	
TyG index	8.6 (0.7)	8.6 (0.8)	8.5 (0.7)	<0.001
TyG-WC	740.3 (662.3, 824.7)	745.3 (656.3, 829.7)	735.3 (660.5, 818.2)	<0.001
TyG-WHtR	4.5 (3.8, 5.1)	4.4 (3.7, 5.2)	4.5 (4.0, 5.0)	<0.001
Age, years old	56.7 (13.1)	55.4 (15.2)	58.8 (9.1)	<0.001
Male (%)	8647 (47.8)	6480 (50.4)	2167 (44.7)	<0.001
SBP, mmHg	124.3 (18.4)	127.7 (22.8)	119.8 (11.2)	<0.001
DBP, mmHg	81.6 (11.3)	86.3 (12.5)	75.1 (7.4)	<0.001
BMI, kg/m²	23.6 (3.1)	23.8 (3.3)	23.3 (1.7)	<0.001
Smoking, n (%)	6013 (34.0)	4551 (35.4)	1462 (30.2)	<0.001

Drinking, n (%)	5935 (33.5)	4346 (33.8)	1589 (32.8)	<0.001
**Education, n (%)**				<0.001
Elementary school	11209 (63.3)	8232 (64.0)	2977 (61.4)	
Middle school	4346 (24.5)	3157 (24.6)	1189 (24.5)	
High school or above	2152 (12.2)	1469 (11.4)	683 (14.1)	
Triglycerides, mmol/L	1.3 (0.7, 2.1)	1.3 (0.7, 2.2)	1.2 (0.8, 1.7)	<0.001
TC, mmol/L	4.4 (3.4, 5.5)	4.5 (3.4, 5.7)	4.2 (3.5, 5.1)	<0.001
HDL-C, mmol/L	1.4 (1.0, 1.7)	1.4 (1.0, 1.8)	1.3 (1.0, 1.6)	<0.001
HbA1c, %	5.6 (1.0)	5.7 (1.1)	5.5 (0.8)	<0.001
FBG, mmol/L	5.6 (1.4)	5.6 (1.5)	5.7 (1.2)	<0.001
Uric acid, μmol/L	299.6 (241.5, 365.7)	311.4 (252.1, 379.3)	297.0 (245.3, 359.6)	<0.001

Q, quartile; TyG, triglyceride-glucose; WHtR, waist to height ratio; WC, waist circumference; SBP, systolic blood pressure; DBP, diastolic blood pressure; BMI, body mass index; TC, total cholesterol; LDL-C, low density lipoprotein cholesterol; HbA1c, glycosylated hemoglobin A1c; FBG, fasting blood glucose.

Supplemental Table S4 Baseline characteristics of participants stratified by outcome in CHNS.

Characteristics	Overall	New-onset hypertension		
		No	Yes	*P* value
n	4600	3389	1211	
TyG index	8.6 (0.7)	8.5 (0.7)	8.7 (0.7)	<0.001
TyG-WC	747.1 (671.6, 830.2)	731.6 (655.8, 813.8)	788.5 (702.6, 872.2)	<0.001
TyG-WHtR	4.5 (4.0, 5.0)	4.4 (3.9, 4.9)	4.6 (4.2, 5.0)	<0.001
Age, years old	48.1 (13.6)	46.7 (13.4)	53.6 (12.5)	<0.001
Male (%)	2058 (44.7)	1468 (43.3)	590 (48.7)	0.001
SBP, mmHg	116.4 (11.2)	114.6 (11.0)	121.4 (10.2)	<0.001
DBP, mmHg	76.0 (7.5)	75.0 (7.6)	78.7 (6.7)	<0.001
BMI, kg/m²	22.9 (3.2)	22.6 (3.1)	23.7 (3.3)	<0.001

Smoking, n (%)	1286 (28.0)	913 (26.9)	373 (30.8)	0.011
Drinking, n (%)	1480 (32.2)	1039 (30.7)	441 (36.4)	<0.001
Urban residence, n (%)	1335 (29.0)	1038 (30.6)	297 (24.5)	<0.001
**Education, n (%)**				<0.001
Illiteracy	937 (20.4)	583 (17.2)	354 (29.2)	
Primary school	927 (20.2)	668 (19.7)	259 (21.4)	
Middle school	2169 (47.2)	1675 (49.4)	494 (40.8)	
Triglycerides, mmol/L	1.2 (0.8, 1.8)	1.1 (0.8, 1.7)	1.3 (0.9, 2.0)	<0.001
TC, mmol/L	4.7 (4.1, 5.4)	4.6 (4.1, 5.3)	4.9 (4.3, 5.5)	<0.001
LDL-C, mmol/L	2.9 (2.3, 3.5)	2.8 (2.3, 3.4)	3.0 (2.4, 3.6)	<0.001
HbA1c, %	5.5 (0.8)	5.5 (0.7)	5.7 (1.0)	<0.001
FBG, mmol/L	5.3 (1.3)	5.2 (1.2)	5.5 (1.5)	<0.001

Uric acid, μmol/L	284.0 (231.0, 346.3)	280.0 (227.0, 342.0)	297.0 (240.5, 361.0)	<0.001

Q, quartile; TyG, triglyceride-glucose; WHtR, waist to height ratio; WC, waist circumference; SBP, systolic blood pressure; DBP, diastolic blood pressure; BMI, body mass index; TC, total cholesterol; LDL-C, low density lipoprotein cholesterol; HbA1c, glycosylated hemoglobin A1c; FBG, fasting blood glucose.

Supplemental Table S5 Baseline characteristics of participants stratified by outcome in CHARLS.

Characteristics	Overall	New-onset hypertension		
		No	Yes	*P* value
n	4849	3637	1212	
TyG index	8.5 (0.7)	8.4 (0.6)	8.7 (0.7)	<0.001
TyG-WC	735.3 (660.5, 818.2)	723.4 (644.3, 801.9)	790.5 (703.6, 873.7)	<0.001
TyG-WHtR	4.5 (4.0, 5.0)	4.3 (3.8, 4.9)	4.7 (4.3, 5.1)	<0.001
Age, years old	58.8 (9.1)	57.5 (9.4)	59.3 (8.8)	<0.001
Male (%)	2167 (44.7)	1611 (44.3)	556 (45.9)	<0.001
SBP, mmHg	119.8 (11.2)	112.7 (12.4)	124.3 (10.2)	<0.001
DBP, mmHg	75.1 (7.4)	74.5 (7.9)	76.7 (6.5)	<0.001
BMI, kg/m²	23.3 (1.7)	22.8 (2.1)	24.7 (1.5)	<0.001

Smoking, n (%)	1462 (30.2)	1032 (28.4)	430 (35.5)	0.011
Drinking, n (%)	1589 (32.8)	1153 (31.7)	436 (36.0)	<0.001
**Education, n (%)**				<0.001
Elementary school	2977 (61.4)	2249 (61.8)	728 (60.1)	
Middle school	1189 (24.5)	868 (23.9)	320 (26.4)	
High school or above	683 (14.1)	502 (13.8)	181 (14.9)	
Triglycerides, mmol/L	1.2 (0.8, 1.7)	1.1 (0.8, 1.7)	1.3 (0.9, 2.0)	<0.001
TC, mmol/L	4.2 (3.5, 5.1)	4.1 (3.7, 4.6)	4.6 (4.0, 5.2)	<0.001
HDL-C, mmol/L	1.3 (1.0, 1.6)	1.4 (1.0, 1.7)	1.2 (0.9, 1.5)	<0.001
HbA1c, %	5.5 (0.8)	5.5 (0.8)	5.6 (1.0)	<0.001
FBG, mmol/L	5.7 (1.2)	5.6 (1.3)	5.9 (1.0)	<0.001
Uric acid, μmol/L	297.0 (245.3, 359.6)	294.4 (240.7, 356.4)	307.7 (249.4, 370.5)	<0.001

Q, quartile; TyG, triglyceride-glucose; WHtR, waist to height ratio; WC, waist circumference; SBP, systolic blood pressure; DBP, diastolic blood pressure; BMI, body mass index; TC, total cholesterol; LDL-C, low density lipoprotein cholesterol; HbA1c, glycosylated hemoglobin A1c; FBG, fasting blood glucose.

Supplemental Table S6 The association of TyG index and its related indices with hypertension after excluding the questionnaire data defining hypertension in Fuqing Cohort.

Variables	Crude model		Model1		Model2	
	OR (95% CI)	*P* value	OR (95% CI)	*P* value	OR (95% CI)	*P* value
**Quartiles of TyG index**						
Q1 (≤8.0)	Ref.		Ref.		Ref.	
Q2-Q4 (>8.0)	1.65 (1.42-1.90)	<0.001	1.23 (1.06-1.40)	<0.001	1.22 (1.06-1.42)	<0.001
Per 1.0 increase	1.37 (1.26-1.48)	<0.001	1.12 (1.04-1.23)	<0.001	1.08 (1.04-1.24)	0.013
**Quartiles of TyG-WHtR index**						
Q1 (≤4.0)	Ref.		Ref.		Ref.	
Q2-Q4 (>4.0)	1.86 (1.45-2.36)	<0.001	1.44 (1.26-1.68)	<0.001	1.42 (1.21-1.65)	<0.001
Per 1.0 increase	1.46 (1.32-1.60)	<0.001	1.21 (1.06-1.39)	<0.001	1.21 (1.05-1.38)	<0.001

**Quartiles of TyG-WC index**						
Q1 (≤634.0)	Ref.		Ref.		Ref.	
Q2-Q4 (>634.0)	1.81 (1.41-2.27)	<0.001	1.40 (1.18-1.64)	<0.001	1.39 (1.17-1.62)	<0.001
Per 100.0 increase	1.38 (1.20-1.58)	<0.001	1.18 (1.07-1.30)	<0.001	1.18 (1.06-1.31)	<0.001

Model 1: adjusted for sex, age, education, urban, BMI, smoking and drinking;

Model 2 (Full model): Model1 and further adjusted for uric acid, HDL-C, TC and HbA1c;

TyG, triglyceride-glucose; WHtR, waist to height ratio; WC, waist circumference; Q, quartile; OR, odds ratio; CI, confidence interval; Ref., Reference.

Supplemental Table S7 The association of TyG index and its related indices with new-onset hypertension after excluding the questionnaire data defining hypertension in CHNS.

Variables	Crude model		Model1		Model2	
	**HR (95% CI)**	***P* value**	**HR (95% CI)**	***P* value**	**HR (95% CI)**	***P* value**
**Quartiles of TyG index**						
Q1 (≤8.0)	Ref.		Ref.		Ref.	
Q2-Q4 (>8.0)	1.66 (1.44-1.92)	<0.001	1.24 (1.07-1.44)	0.004	1.24 (1.06-1.45)	0.007
Per 1.0 increase	1.38 (1.28-1.48)	<0.001	1.12 (1.03-1.21)	0.007	1.05 (1.03-1.28)	0.012
**Quartiles of TyG-WHtR index**						
Q1 (≤4.0)	Ref.		Ref.		Ref.	
Q2-Q4 (>4.0)	2.06 (1.54-2.76)	<0.001	1.77 (1.49-2.09)	<0.001	1.74 (1.32-2.30)	<0.001
Per 1.0 increase	1.56 (1.42-1.72)	<0.001	1.25 (1.06-1.48)	<0.001	1.22 (0.97-1.53)	<0.001

**Quartiles of TyG-WC index**						
Q1 (≤634.0)	Ref.		Ref.		Ref.	
Q2-Q4 (>634.0)	1.91 (1.51-2.41)	<0.001	1.82 (1.19-2.68)	<0.001	1.70 (1.32-2.14)	<0.001
Per 100.0 increase	1.48 (1.30-1.69)	<0.001	1.20 (1.08-1.32)	<0.001	1.18 (1.07-1.30)	<0.001

Model 1: adjusted for sex, age, education, urban, BMI, baseline SBP and DBP, smoking and drinking;

Model 2 (Full model): Model1 and further adjusted for uric acid, HDL-C, TC and HbA1c;

TyG, triglyceride-glucose; WHtR, waist to height ratio; WC, waist circumference; Q, quartile; HR, hazard ratio; CI, confidence interval; Ref., Reference.

Supplemental Table S8 The association of TyG index and its related indices with hypertension defined by the novel diagnostic criteria (SBP/DBP: 130/80) in Fuqing Cohort.

Variables	Crude model		Model1		Model2	
	**OR (95% CI)**	***P* value**	**OR (95% CI)**	***P* value**	**OR (95% CI)**	***P* value**
**Quartiles of TyG index**						
Q1 (≤8.0)	Ref.		Ref.		Ref.	
Q2-Q4 (>8.0)	1.62 (1.34-1.82)	<0.001	1.23 (1.08-1.40)	<0.001	1.19 (1.03-1.36)	<0.001
Per 1.0 increase	1.32 (1.23-1.45)	<0.001	1.11 (1.03-1.28)	<0.001	1.08 (1.04-1.24)	0.003
**Quartiles of TyG-WHtR index**						
Q1 (≤4.0)	Ref.		Ref.		Ref.	
Q2-Q4 (>4.0)	1.78 (1.42-2.18)	<0.001	1.36 (1.22-1.64)	<0.001	1.35 (1.20-1.50)	<0.001
Per 1.0 increase	1.36 (1.18-1.60)	<0.001	1.22 (1.12-1.34)	<0.001	1.21 (1.05-1.47)	<0.001

**Quartiles of TyG-WC index**						
Q1 (≤635.0)	Ref.		Ref.		Ref.	
Q2-Q4 (>635.0)	1.75 (1.36-2.20)	<0.001	1.40 (1.12-1.70)	<0.001	1.38 (1.11-1.63)	<0.001
Per 100.0 increase	1.37 (1.20-1.55)	<0.001	1.18 (1.05-1.30)	<0.001	1.17 (1.04-1.30)	<0.001

Model 1: adjusted for sex, age, education, urban, BMI, smoking and drinking;

Model 2 (Full model): Model1 and further adjusted for uric acid, HDL-C, TC and HbA1c;

TyG, triglyceride-glucose; WHtR, waist to height ratio; WC, waist circumference; Q, quartile; OR, odds ratio; CI, confidence interval; Ref., Reference.

Supplemental Table S9 The association of TyG index and its related indices with new-onset hypertension defined by the novel diagnostic criteria (SBP/DBP: 130/80) in CNHS.

Variables	Crude model		Model1		Model2	
	**HR (95% CI)**	***P* value**	**HR (95% CI)**	***P* value**	**HR (95% CI)**	***P* value**
**Quartiles of TyG index**						
Q1 (≤8.0)	Ref.		Ref.		Ref.	
Q2-Q4 (>8.0)	1.42 (1.24-1.62)	<0.001	1.15 (1.01-1.32)	0.041	1.09 (0.94-1.26)	0.26
Per 1.0 increase	1.32 (1.23-1.43)	<0.001	1.09 (1.00-1.19)	0.045	0.98 (0.87-1.09)	0.684
**Quartiles of TyG-WHtR index**						
Q1 (≤4.0)	Ref.		Ref.		Ref.	
Q2-Q4 (>4.0)	1.78 (1.52-2.08)	<0.001	1.33 (1.13-1.56)	0.001	1.32 (1.12-1.57)	0.001
Per 1.0 increase	1.39 (1.28-1.50)	<0.001	1.13 (1.04-1.24)	0.005	1.15 (1.02-1.29)	0.024

**Quartiles of TyG-WC index**						
Q1 (≤635.0)	Ref.		Ref.		Ref.	
Q2-Q4 (>635.0)	1.55 (1.26-1.90)	<0.001	1.51 (1.23-1.86)	<0.001	1.34 (1.08-1.67)	<0.001
Per 100.0 increase	1.37 (1.30-1.45)	<0.001	1.09 (1.00-1.18)	0.052	1.14 (1.02-1.26)	0.027

Model 1: adjusted for sex, age, education, urban, BMI, baseline SBP and DBP, smoking and drinking;

Model 2 (Full model): Model1 and further adjusted for uric acid, HDL-C, TC and HbA1c;

TyG, triglyceride-glucose; WHtR, waist to height ratio; WC, waist circumference; Q, quartile; HR, hazard ratio; CI, confidence interval; Ref., Reference.

Supplemental Table S10 The association of TyG index and its related indices with new-onset hypertension defined by the novel diagnostic criteria (SBP/DBP: 130/80) in CHARLS.

Variables	Crude model		Model1		Model2	
	**HR (95% CI)**	***P* value**	**HR (95% CI)**	***P* value**	**HR (95% CI)**	***P* value**
**Quartiles of TyG index**						
Q1 (≤8.1)	Ref.		Ref.		Ref.	
Q2-Q4 (>8.1)	1.38 (1.14-1.70)	<0.001	1.17 (1.02-1.33)	0.035	1.19 (1.05-1.36)	0.181
Per 1.0 increase	1.28 (1.15-1.42)	<0.001	1.08 (1.00-1.18)	0.045	1.09 (0.98-1.20)	0.511
**Quartiles of TyG-WHtR index**						
Q1 (≤4.0)	Ref.		Ref.		Ref.	
Q2-Q4 (>4.0)	1.88 (1.45-2.20)	<0.001	1.40 (1.12-1.72)	0.001	1.36 (1.16-1.58)	0.001
Per 1.0 increase	1.41 (1.25-1.60)	<0.001	1.15 (1.02-1.28)	<0.001	1.17 (1.03-1.29)	<0.001
**Quartiles of TyG-WC index**						
Q1 (≤635.0)	Ref.		Ref.		Ref.	
Q2-Q4 (>635.0)	1.70 (1.25-1.98)	<0.001	1.31 (1.14-1.52)	<0.001	1.27 (1.05-1.49)	<0.001
Per 100.0 increase	1.38 (1.15-1.65)	<0.001	1.15 (1.02-1.28)	0.038	1.13 (1.01-1.26)	0.031

Model 1: adjusted for sex, age, education, urban, BMI, baseline SBP and DBP, smoking and drinking;

Model 2 (Full model): Model1 and further adjusted for uric acid, HDL-C, TC and HbA1c;

TyG, triglyceride-glucose; WHtR, waist to height ratio; WC, waist circumference; Q, quartile; HR, hazard ratio; CI, confidence interval; Ref., Reference.
